# Protozoan and Microbial Pathogens of House Cats in the Province of Tekirdag in Western Turkey

**DOI:** 10.3390/pathogens10091114

**Published:** 2021-08-31

**Authors:** Mustafa Necati Muz, Serkan Erat, Kosta Y. Mumcuoglu

**Affiliations:** 1 Department of Parasitology, Faculty of Veterinary Medicine, University of Namik Kemal, Tekirdag 59000, Turkey; 2 Department of Animal Breeding and Husbandry, Faculty of Veterinary Medicine, Kirikkale University, Kirikkale 71450, Turkey; serat@kku.edu.tr; 3 Parasitology Unit, Department of Microbiology and Molecular Genetics, The Kuvin Center for the Study of Infectious and Tropical Diseases, Hadassah Medical School, The Hebrew University, Jerusalem 91120, Israel; kostasm@ekmd.huji.ac.il

**Keywords:** cat, One Health, zoonoses, PCR, Turkey

## Abstract

Domestic felines’ re-emerging infectious and neglected zoonotic diseases are a significant focus of global “One Health” efforts. This study aimed to rapidly diagnose 14 pathogens, including zoonoses by using PCR primers in 167 client-owned symptomatic cats, routinely accepted to the Veterinary Clinics of Tekirdag. The prevalence of pathogens investigated were as follows: *Babesia canis canis* (24%), *Babesia microti* (2.4%), *Hepatozoon felis* (10.8%), *Cytauxzoon felis* (6.6%), *Bartonella henselae* (40.1%), *Anaplasma platys* (30.5%), *Anaplasma phagocytophilum* (7.2%), *Rickettsia felis* (26.3%), *Borrelia burgdorferi* (21%), and hemotropic *Mycoplasma sp.* (11.4%). There was a significant difference between the prevalence of the pathogens (*χ*^2^ = 152.26, df = 9, *p* < 0.001). There was also a statistical difference between the gender of the cats in terms of the prevalence of all pathogens considered together (*χ*^2^ = 4.80, df = 1, *p* = 0.028), where the female cats showed a higher prevalence. This was not the case for the different age groups (*χ*^2^ = 2.92, df = 1, *p* = 0.088). The lowest infection was observed for *B. microti* (*p* < 0.001), while the highest infection was observed for *B. henselae* (*p* < 0.01). *Leishmania donovani*, *Plasmodium* spp., *Ehrlichia chaffeensis*, and *Neoehrlichia mikurensis* PCR test results were negative in all samples. In conclusion, house cats of Tekirdag are apparently highly susceptible to some neglected zoonoses important for “One Health”, and their prevalence in the region is most probably underestimated. Hence, applying PCR tests to assist fast clinic diagnosis in routine, may be an efficient option to protect the public as well as the cats from severe diseases.

## 1. Introduction

In Turkey, where about 84 million people live, almost 19% of the population have cats, however, the number of stray cats is not known [1]. Cats are hosts of ectoparasites such as fleas, ticks, mosquitoes, and accordingly, act as reservoirs for pathogens of medical and veterinary importance, especially because of their close association with humans. In this context, infected cats could be one of the sources of zoonotic diseases, and neglected epidemiological monitoring might be the cause of increased public health and veterinary concerns [2,3].

There are reports about the increased infections by the tick-borne, zoonotic *Babesia microti* in mammals and humans in endemic areas, which is a notifiable human disease also in the USA [4]. Pennisi et al. [5] suggested that cats could be a reservoir of *B. microti*. Species such as *Babesia canis canis* usually infecting dogs, may also be found in cats [6]. So far, *B. canis canis* and *B. microti* have not been reported in cats from Turkey, but the presence of their DNA in vector ticks and rodents is known. Oral transmission of *B. microti* in mice was confirmed by experimental ingestion of infected blood and by cannibalism [7], while *Rhipicephalus sanguineus, Dermacentor reticulatus*, and *Dermacentor marginatus*, known *B. canis canis* vectors, have been identified in the country [8].

According to Baneth et al. [9], *Hepatozoon felis* is a tick-borne disease, however, it can also be transmitted transplacentally. Unlike wild cat species, the only species to invade domestic cats’ heart muscles is *H. felis,* however, *H. felis* cases in domestic cats were rarely reported. In Israel, schizonts of *Hepatozoon sp*. have been detected in heart tissues of 100 cats examined in autopsies [9].

*Cytauxzoon felis* is one of the blood-borne cat hemoprotozoa. Cats are the main reservoir of *C. felis*, while *Dermacentor variabilis* and *Amblyomma americanum* are confirmed vectors of this pathogen [10]. Both tick species are not present in Turkey, while *Ixodes ricinus* serves as a vector of this pathogen [11].

Cats are the principal reservoirs of *Bartonella henselae*, while the main vector is the cat-flea, *Ctenocephalides felis* [12]. The transstadial transmission of *B. henselae* in *R. sanguineus* is confirmed [13], while *I. ricinus* is an accepted vector of this pathogen [14]. The wounds generated by cat scratches may cause human infections if contaminated with flea feces. Bartonellosis is known as a self-limiting zoonotic disease and can be persisted in reservoir cats. Studies indicated that more than 50% of carrier cats are asymptomatic [12].

*Anaplasma platys,* which is among the vector-mediated blood parasites of domestic cats may result in a mild illness in the animals and with non-specific symptoms in humans. The transstadial, transovarial, and horizontal transmission of *A. platys* by *R. sanguineus* is confirmed, while *I. ricinus* is a vector of *Anaplasma phagocytophilum* [15]. Reporting human anaplasmosis is notifiable in the USA [16]. Feline anaplasmosis may be considered as a neglected, re-emerging zoonotic disease [17]. 

Zoonotic flea-borne spotted fever caused by *Rickettsia felis* has been reported, particularly from port cities, coastal areas with increased reservoirs and vectors. Dogs can act as natural mammalian reservoir hosts for this zoonosis [18]. *Rickettsia felis* was identified in different countries from *C. felis, Ctenocephalides canis, Pulex irritans, Archeopsylla erinacei, Xenopsylla cheopis, Leptopsylla segnis, R. sanguineus, Rhipicephalus bursa*, and *Pulex irritans*. Though some of these species exist in the study area, there are no reports about their vector competencies [11].

Lyme disease (LD) is one of the most frequently reported tick-mediated infectious diseases in the Northern Hemisphere’s moderate climatic regions. The main vectors for this disease are ticks of the genus Ixodes. The zoonotic LD is still medically neglected in asymptomatic cats. Since the laboratory results and symptoms of Lyme disease are not specific, and difficult to diagnose by clinical examination alone, highly sensitive diagnostic methods such as PCR supported with specific primers, should be preferred [19,20]. 

Cats usually have latent Mycoplasma infections. Chronic infections are usually not associated with marked clinical signs, although infections by reactivation may be possible. The way mycoplasmas are transmitted among cats is not known, though the role of vector arthropods and aggressive feline interactions may be conceivable. Specific diagnosis is currently reliant on detecting mycoplasmic DNA using PCR, which is more sensitive than staining. Immunodeficient humans may rarely become infected with feline-originated *Candidatus* Mycoplasma haemominutum, *Candidatus* Mycoplasma turicensis, and *Candidatus* Mycoplasma haemofelis species [21]. 

The monocytotropic human ehrlichiosis caused by *Ehrlichia chaffeensis* was first described in the USA, while thereafter domestic cat-originated *E. chaffeensis* cases were reported in the USA and Brazil [17]. Leishmaniasis vectored by the genus *Phlebotomus* is considered a neglected zoonosis under “category one” diseases, endemic in close to 100 countries. With an annual case prevalence of 1.5 to 2 million and 70,000 deaths per year, measures should be taken from the global “One Health” perspective. Almost 2,000 autochthonous cutaneous leishmaniasis (CL) cases are detected annually in Turkey [22]. Pennisi et al. reported fifty cases of cat leishmaniosis in European countries between 1989 and 2014 [23]. In Italy, 286 healthy cats were examined and 30.8% of them were seropositive for anti-Leishmania IgG, while 15.7% of them were positive when their conjunctival swaps were examined by nested PCR [24], while Morelli et al. [25] examined 269 cats from Italy and Greece and 3% of them were seropositive for anti-Leishmania infantum IgG. Though few clinical cases of leishmaniasis in cats were reported, it is possible that these animals could be under given circumstances be a reservoir for this zoonotic disease [26]. The first molecular identification test for leishmania in Turkish domestic cats dates back to 2015 [27]. The medical and veterinary significance of the newly discovered tick-borne agent *Neoehrlichia mikurensis* is still not clear. It is known to infect dogs but not cats. The presence of *N. mikurensis* was confirmed in *Ixodes ricinus* collected from Eastern Romania [28].

To the best of our knowledge, there are no molecular records related to *B. microti*, *B. canis canis*, *C. felis*, *H. felis*, *R. felis*, *A. phagocytophilum*, *A. platys*, and *Borrelia burgdorferi* in domestic cats in Turkey. Moreover, little is known about the infectious agents circulating in house cats in the research area. Veterinarians need quick and reliable identification of the pathogens relevant to animal health before they can initiate an effective treatment. The aim of this study was to investigate the prevalence of vector-borne pathogens of medical and veterinary significance in house cats in the province of Tekirdag, by using a rapid, species-specific PCR test.

## 2. Method

### 2.1. Sampling Area

**Figure 1:**
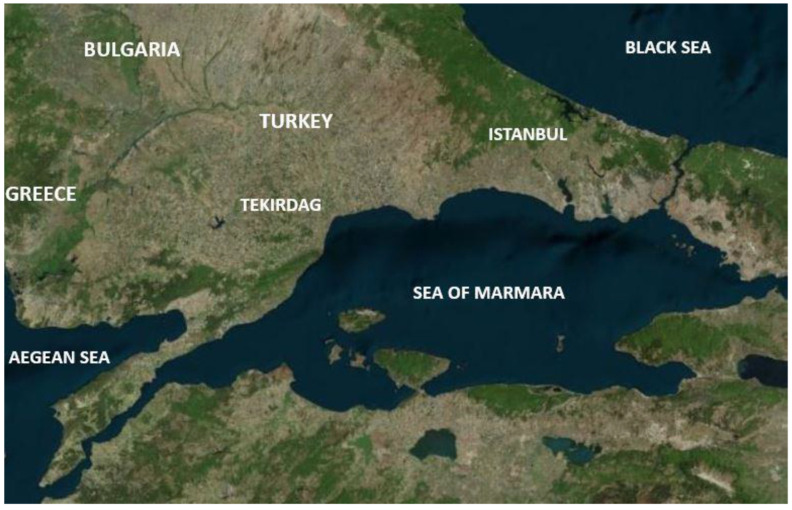
The geographical location of the sampling area in Northwestern Turkey.

The population of the Tekirdag Province is approximately 1.2 million, and its geographical location is between Northern Marmara and the Western Black Sea (26°43′–28°08′ D/40°36′–41°31′ N) (Figure 1). The average altitude is 37 m, the annual precipitation is 590 mm, and the average temperature is 14 °C.

### 2.2. Animals and Sample Collection

Blood samples (n: 167) were taken from cats suffering from symptoms such as weight loss, fever, hematological abnormalities, and lymphadenopathy and admitted to a Veterinary Clinic between 2017 and 2021. Cat peripheral blood samples were collected in EDTA-coated tubes by venipuncture. Samples were centrifuged at 400× *g* for 10 min, (Rotina 380R, Hettich, Tuttlingen, Germany) the buffy coat was aspirated, and the erythrocytes were aliquoted and preserved at –80 °C (U700, Daihan, Korea). The age and gender of the animals were recorded. The research was conducted with the permission of the Tekirdag Namik Kemal University Ethics Committee (Approval numbers: T2021/576/07 and 2017/07/01). The animals were not examined for ectoparasites.

### 2.3. DNA Extraction

Blood specimens were thawed, vortexed, and 200 µL was used for DNA extraction. The commercial extraction kit (GeneJET Genomic DNA Extraction Kit, Thermo, Lithuania) was used for this purpose. DNA was extracted following the commercial manufacturer’s recommendations from feline blood specimens. DNA samples were tested using the PCR (T100, BioRad, Singapore) protocols [12,21,29,30,31,32,33,34,35,36,37,38,39,40]. The samples were examined for the presence of protozoa such as *B. microti*, *B. canis canis*, *H. felis*, *C. felis, Plasmodium* spp., *Leishmania donovani*, and bacteria such as *A. phagocytophilum*, *A. platys*, *E. chaffeensis*, *R. felis*, *B. henselae*, *B. burgdorferi*, *N. mikurensis*, and hemotropic *Mycoplasma* spp. The final PCR reaction volume was 25 µL, consisting of 10× Taq buffer (Thermo, Lithuania), 4 mM MgCl_2_, 400 nM dNTP mix, 400 nM each forward/reverse primer, and 2u Taq DNA polymerase (Thermo, Lithuania). The synthesized oligonucleotides previously confirmed primers targeting specific gene regions of the tested pathogens were used (Table 1). DNA samples isolated from previous clinical specimens were used for both PCR optimization and positive control, and water was used as the negative control. The cycling conditions applied in the PCR test were the same for each pathogen, except for the annealing conditions. Initial denaturation was 5 min at 95 °C, 40 cycles for 30 s at 95 °C for denaturation, the primer specific annealing temperature for 30 s which are listed in Table 1, and 72 °C for 60 s for extension followed by the final extension at 72 °C 10 min. 

### 2.4. Agarose Gel Electrophoresis

Low-melting agarose was used for matrix gel (containing 0.5 μg/mL EtBr in a density of 1.5% matrix) to run over the "PCR amplimers" at 100 V for 45 min. The bands were screened via a UV transilluminator camera attachment (WiseDoc WGD-30, Daihan, Korea).

### 2.5. Statistical Analysis

The chi-square (*χ*^2^) analysis was performed to find gender and age group differences in pathogen prevalence. Fisher’s exact test was used when the expected values were less than 5 in 2 × 2 crosstabs. Adjusted residual (z-scores) were used to calculate *p* values to see if there were differences between pathogens in terms of prevalence. For this, the new critical z score was calculated as –3.02 and the critical *p*-value was 0.0025 after Bonferroni correction. Spearman rank correlations (rho) were calculated to see if there was a relationship between pathogens. All the analyses were performed using IBM SPSS Statistics for Windows, v 25.0 (Armonk, NY: IBM Corp.).

## 3. Results

Overall, 167 mix-bred, short-hair cats (82 females and 85 males) which were brought by their owner to our clinics, were examined. Overall, 88 cats were aged >1 year while 79 cats were aged ≤1 year. The following pathogens were recorded in the blood of the cats: *B. canis canis* (24%), *B. microti* (2.4%), *H. felis* (10.8%), *C. felis* (6.6%), *B. henselae* (40.1%), *A. platys* (30.5%), *R. felis* (26.3%), *B. burgdorferi* (21%), hemotropic *Mycoplasma sp.* (11.4%), and *A. phagocytophilum* (7.2%) (Table 1).

The chi-squared test showed that there was a significant difference in the prevalence of the different pathogens (*χ*^2^ = 152.26, df = 9, *p* < 0.001). The lowest infection was observed for *B. microti* (*p* < 0.001), while the highest for *B. henselae* (*p* < 0.01) (Table 2). 

There was no difference between male and female cats in terms of the prevalence of the pathogens alone (*p* > 0.05) (Table 3).

There was also no difference between age groups in terms of pathogen prevalence (*p* > 0.05) except for *A. phagocytophilum* for which >1-year old cats showed higher prevalence (*χ*^2^ = 7.88, df = 1, *p* = 0.005) (Table 4).

There was a statistical difference between male and female cats in terms of prevalence when all pathogens were considered together (*χ*^2^ = 4.80, df = 1, *p* = 0.028), in which case female cats had a higher prevalence of pathogens than males. This was, however, not the case for the two age groups (*χ*^2^ = 2.92. df = 1. *p* = 0.088) (Table 5).

A significant correlation was observed between *B. microti* and *B. canis canis* (rho = 0.28, *p* < 0.001); between *B. canis canis* and *B. burgdorferi* (rho = 0.23, p = 0.003); between *C. felis* and *A. phagocytophilum* (rho = 0.21, *p* = 0.007); between *A. platys* and *B henselae* (rho = 0.25, *p* = 0.001); between *R. felis* and hemotropic *Mycoplasma sp.* (rho = 0.17, *p* = 0.027); as well as between *B. henselae* and *B. burgdorferi* (rho = 0.24, *p* = 0.002) (Table 6).

**Figure 2:**
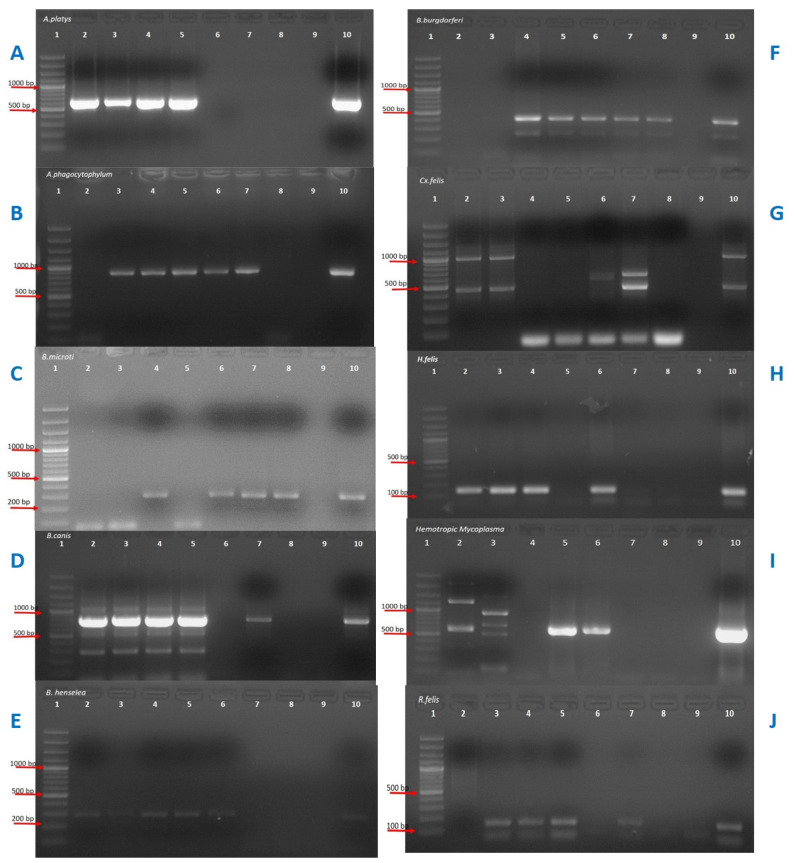
The view of agarose gel electrophoresis of pathogen-specified PCR results. (**A**) line 1; DNA ladder, lines 6–8; negative samples, lines 2–5; positive samples, line 9; negative control, line 10; positive control for *A. platys*. (**B**) line 1; DNA ladder, lines 2, 8; negative samples, lines 3–7 positive samples, line 9; negative control, line 10; positive control for *A. phagocytophilum*. (**C**) line 1; DNA ladder, lines 2, 3, 5; negative samples, lines 4, 6–8; positive samples, line 9; negative control, line 10; positive control for *B. microti.* (**D**) line 1; DNA ladder, lines 6, 8; negative samples, lines 2–5, 7; positive samples, line 9; negative control, line 10; positive control for *B. canis canis.* (**E**) line 1; DNA ladder, lines 7–8; negative samples, lines 2–6; positive samples, line 9; negative control, line 10; positive control for *B. henselae.* (**F**) line1; DNA ladder, lines 2–3; negative samples, lines 4–8; positive samples, line 9; negative control, line 10; positive control for *B. burgdorferi.* (**G**) line 1; DNA ladder, lines 4–6, 8; negative samples, lines 2, 3, 7; positive samples, line 9; negative control, line 10; positive control for *Cx. felis.* (**H**) line 1; DNA ladder, lines 2–4, 6–8; positive samples, line 9; negative control, line 10; positive control for *H. felis.* (**I**) line 1; DNA ladder, lines 3, 4, 7, 8; negative samples, lines 2, 5, 6; positive samples, line 9; negative control, line 10; positive control for Hemotropic Mycoplasma. (**J**) line 1; DNA ladder, lines 2, 6, 8; negative samples, lines 3–5, 7; positive samples, line 9; negative control, line 10; positive control for *R. felis*.

The view of agarose gel electrophoresis of pathogen-specified PCR results can be seen in Figure 2.

## 4. Discussion

In the present study, the following pathogens were identified molecularly in client owned cats: *B. microti*, *B. canis canis*, *H. felis*, *C. felis*, *A. platys*, *A. phagocytophilum*, *B. henselae*, *R. felis*, *B. burgdorferi*, and hemotropic *Mycoplasma sp*., while none of the cats was positive for *Leishmania donovani*, *Plasmodium* spp. and *N. mikurensis*. It should be stressed, however, that blood is not a good target to detect *Leishmania* parasites (24).

*Babesia microti* has rarely been reported in cats, whereas *B. canis canis* is rather reported in dogs around the world, but no feline cases of *B. canis canis* have been reported in Turkey. Previous studies confirmed *B. microti* DNA presence both in *I. ricinus* and *Hyalomma marginatum* found in the country [41]. In the present study, *B. microti* and *B. canis canis* DNA was detected in four and 24 out of 167 samples examined, respectively. In Italy, two out of 260 cats in Milan and six out of 23 cats in Sicily were positive for this pathogen. In Pakistan, *B. microti* was detected in 21 of 159 cats tested by PCR, while *B. canis* was detected in 1.3% of 320 tested cats from Portugal. *Babesia canis canis* was detected by PCR testing in three out of 30 cats sampled from Spain and Portugal [42]. In Turkey, *B. canis canis* was found to be present in 12% of 400 tested dogs [43]. Since the cats are often closely in contact with humans and rodents they can act as a reservoir between wildlife pathogens. *Babesia microti* PCR positivity was reported in 5.8% of the 536 rodents caught between 2010 and 2012 in Bartin and Giresun Provinces of Turkey [41]. A total of 322 blood samples were collected from individuals with tick bites in the Province of Corum, out of which 0.93% were positive for *B. microti* [44]. *Babesia microti* seropositivity was detected in 16 out of 149 humans with a history of tick bites in Van Province and in 6.23% of 273 individuals living in the Black Sea region [45].

While *Hepatozoon felis* DNA was detected in 10.7% of tested domestic cats in this study, the percentage of *H. felis* infected cats was found to be 25% in Cyprus [46], 20.6% in Italy [47], 16% in Spain [48], and 15.6% in Portugal [49]. In Turkey, Tuna et al. reported Hepatozoon DNA in tick-free domestic cats [50], while Orkun et al. detected *Hepatozoon sp.* DNA in 49.5% of the 103 tested shelter dogs in Ankara, out of which 86% were positive for *Hepatozoon canis* and 13.7% for *Hepatozoon* spp., while specimens of *R. sanguineus* collected from a Hepatozoon DNA negative dog was positive for *H. felis* [51]. In addition, *H. felis* DNA was determined in *Haemaphysalis parva* specimens collected in the Ankara region, as well as in 0.31% of *Hyalomma sp.* [51] and in 3% of the 34 *Rhipicephalus turanicus* collected from humans living in Corum Province [44].

In the present study, *C. felis* was detected in 6.6% of the sampled cats. The molecular prevalence of *C. felis* in domestic cats ranged between 3.4% and 15.6% in Arkansas, Missouri, and Oklahoma [52]; it was 21.5% in the Yunan Province of China [53], and 19% in Iran [54]. An earlier hematoscopic study conducted in Turkey suggested a 7.5% positivity in cats [55]. 

The molecular prevalence of cat bartonellosis in the present study was 40.1%. Earlier in the country, PCR tests confirmed the presence of *B. henselae* in cats of Ankara (8.2% to 18.6%) [56], Istanbul (28.1%) [57], and Izmir (8%) [58], while *B. henselae* seropositivity was 34.4% of 186 cats examined in Konya [59], 41.3% in Bursa, 33.9% in Adana, and 27.5% in Aydin [60]. In the Western Aegean region of the country, *B. henselae* IgG seropositivity was higher in pet cat and pet dog owners than in those who were caring for stray cats and stray dogs in their neighborhood (26.5% and 6.8%, respectively) [61]. In addition, the seroprevalence in veterinarians and cattle herders was 30% and 12.5%, respectively, in the Aydin and Denizli Provinces [62]. In Denizli, 6% of 800 blood donors were *B. henselae* seropositive in 2009 [63].

The molecular prevalence of cat bartonellosis ranged from 0.5% to 97.3% in Brazil [64] 38.3% to 80% in Spain [65], and was 15.6% in Israel [66]. The seropositivity of *B. henselae* in Dutch cats ranged from 50% to 56% [67], 18% in Italy [68], and 58.8% in Greece [69]. *Bartonella henselae* has been reported in 45% of veterinarians operating in Poland, and in 53.3% of those keeping domestic cats [70]. *B. henselae* was detected in 3.087 individuals in Israel between the years 1991–2016 [71].

In the USA, 51% of the cats were seropositive for *B. henselae* [72]. In this country, an average of 24,000 cat-scratch cases are detected each year in humans and about 2000 patients are hospitalized [73]. *Bartonella henselae* IgG seropositivity was detected in 61.6% of 608 healthy individuals in Italy [74]. It has been reported that *B. henselae* bacteremia is most common in under one-year-old cats, and less common in older cats [75]. In the current study, this could not be confirmed, as no differences were found between less and more than one-year-old cats. *Borrelia burgdorferi*, the causative agent of Lyme disease, was detected in 21% of the animals examined in the present study. Earlier in Turkey, *B. burgdorferi* DNA was detected in 38.7% of *I. ricinus* examined in the Istanbul region, with 11.4% of those in Kirklareli [76]. This pathogen was also detected in *Hyalomma aegyptium* collected from Thrace forestry [77], as well as in a Saint-Bernard dog from Istanbul [78]. The Lyme agent was previously detected in *Haemaphysalis parva* in Ankara [79] and *I. ricinus* infesting humans around the present study area [80].

*Borrelia burgdorferi* DNA was detected in ticks collected from cats in Germany and France [81] and in 25.7% of *Ixodes scapularis* ticks collected from cats at veterinary practices in the USA [82]. The seropositivity for LD in Trabzon Province of Turkey ranged between 0.9–14.5% [83]. Between 2010 and 2018 in the USA, 476,000 LD cases were diagnosed annually. According to the most recent statement of the European Parliament on Lyme disease, it is estimated that approximately 850,000 cases of LD occur each year while the actual numbers are thought to be much higher [84]. In northern parts of the United States, seroprevalence ranged from 13% to 47% in cats [85] and was 15.8% in the Czech Republic [86].

In the present study, *R. felis* is being reported for the first time in cats of Turkey. Earlier, *R. felis* was detected in *Rhipicephalus bursa* ticks collected from humans in Istanbul [76]. In the USA, *R. felis* DNA was detected in 0.5% of cats (n: 722) and in 0.4% of the dogs (n: 777) [87]. Seropositivity to *R. felis* in cats has been reported from the US, Chile, Italy, and Taiwan [24,88]. This pathogen was reported to be found in *I. Ricinus*. The seropositivity rate of *A. phagocytophilum* in humans from Turkey was 10.6% in Sinop, 5.77% in Tokat [89], 8% in Antalya [90], and 25% in Edirne [91]. The number of human anaplasmosis cases in the USA increased from 348 in 2000 to 5,762 in 2017 [92]. In Poland, *A. phagocytophilum* DNA was detected in 120 out of 1,375 patients with a history of tick bites, and a total of 32 human cases of *A. phagocytophilum* in a decade-long study in Europe also describes its prevalence [93,94]. Earlier, anaplasmosis was reported in Turkey from ruminants, equines, carnivores (including dogs), and humans, but never from domestic cats [44,95]. *Anaplasma platys* DNA was detected in dogs as well as in *R. sanguineus* and *R. turanicus* specimens. *Anaplasma phagocytophilum* DNA was detected in *I. ricinus* samples collected from Istanbul (2.7%), Kirklareli (17.5%), and the Black Sea region (11.6%) [76,87,96,97]. In addition, *A. phagocytophilum* DNA has been detected in *Rhipicephalus bursa* and *H. parva* samples [87]. In Brazil, 13.2% of 91 cats were found positive for *A. platys* in the PCR test, while the seroprevalence of *A. phagocytophilum* ranged between 2–8% in domestic cats in the Mediterranean coastal provinces of Spain and Italy, 23.1% in northern Italy, 0.4% in Germany, 1.7% in England, 5.4% in Southern Portugal, 0.9% in Korea, and 38% in the northeast USA [88,98,99,100]. *Anaplasma platys* PCR positivity in cats of the present study was 30.5%, while this of *A. phagocytophilum* was found to be 7.2%. 

*Rickettsia felis* was reported in *I. scapularis* in Romania and in *D. variabilis* in USA [101,102,103]. *Rickettsia felis* was also detected in flea samples collected from domestic animals, including cats, in Greece [103], and in *C. felis* and *C. canis* collected from dogs and cats in Italy [104], from *Archaeopsylla erinacei*, *Ctenophthalmus baeticus boisseauorum*, and *C. felis* in Spain, France, and in *Ixodes hexagonus* in Italy [105,106]. Individuals who sleep near flea-infested reservoir cats and dogs, or pets belonging to owners traveling in endemic areas, are at higher risk of becoming infected with rickettsiosis. Interestingly, exposure to *R. felis* was unexpectedly high (16%) among Australian veterinarians [107]. Human *R. felis* infections were also reported in the USA, Australia, New Zealand, Israel, Laos, Thailand Taiwan, South Korea, Tunisia, Kenya, Senegal, Mexico, Brazil, France, Germany, Spain, and Sweden [108].

Overall, 11.4% of the cats in the study area were positive for hemotropic Mycoplasma. The first local case in the country was detected in 1991 in Istanbul [109]. The prevalence of hemotropic Mycoplasma in cats was 7.7% in Bursa, 17.5% in Izmir, 17.5% in Antalya, 30.8% in Ankara, 95% in Kayseri, 14.9% in Van, and 19.3% in Istanbul [110,111]. The DNA of this pathogen was detected in 30% of domestic cats in Spain [6], 17.2% in Serbia [112], 21.6% in Romania [113], 31% in New Zealand [114], 26% in Cyprus [46], and 13.2% in Italy [115]. In the current study, no significant difference was found between pathogen prevalence and age distribution, with the exception for *A. phagocytophilum*, where animals over one-year-old had a significantly higher prevalence as compared to less than one-year-old animals. Female cats in this study had a higher prevalence of pathogens than the males. The fact that a veterinarian in the USA was co-infected with *A. platys, B. henselae*, and *Mycobacterium haematoparvum* indicates the public health importance that is threatened by these parasites. It is important to stress that in stray cats, the infestation rate with pathogens observed in the present study could be higher as compared to symptomatic indoor cats, while additional pathogens can be expected to be detected in stray cats.

The fact that different positive percentages regarding a parasite were obtained in different countries, might be explained that the studies were conducted in different years, seasons, geo-climatic areas, domestic versus street cats, healthy versus symptomatic cats, and the examination techniques used.

## 5. Conclusions

Where cats exist, so do neglected zoonotic diseases, hence, re-emerging infectious agents and neglected feline zoonoses are a growing concern in the “One Health” approach. The present study shows that a high percentage of house cats were positive to one or more pathogens out of the 10 that were examined in the province of Tekirdag, in the European side of Turkey. The *B. microti, B. canis canis, H. felis, C. felis, B. henselae, A. platys, A. phagocytophilum, R. felis*, and *B. burgdorferi* were reported in house cats of the region. Molecular techniques such as PCR can be used for the quick and reliable diagnosis of a pathogenic agent and thus facilitate the appropriate treatment by veterinarians. A species-specific primer used in PCR assays can be useful to distinguish closely related species at the subspecies level and may help to support the treatment protocol. The present study on feline zoonotic endemicity in northwestern Turkey could form a basis for the cognitive increase in “One Health” awareness. Sustainable cooperation of veterinarians, public health officials, and local authorities may be of paramount importance for the detection, monitoring, and control of pathogens of medical and veterinary importance. Because of the risk of vector-borne infection for both domestic cats and public health, vitally important pathogens need to be monitored periodically and pets should be treated for ectoparasites. Zoonoses and vector-borne infectious with cat pathogens may reason significant health risks that cannot be neglected. Since some of the above-mentioned pathogens are zoonotic, medical awareness should be provided to pet owners, veterinarians, and healthcare professionals.

## Figures and Tables

**Table 1:** The primer pairs used in PCR protocols in this study. F: Forward primer, R: Reverse primer.

No	Identified Pathogens	Primer Sequences (5′–3′)	Annealing/°C	Product Size bp	Reference
1	*Babesia microti*	F: ATAGGTCAGAAACTTGAATGATACA R: CTTAGTATAAGCTTTTATACAGC	55	238	[29]
2	*Babesia canis canis*	F: GTGAACCTTATCACTTAAAGG R: CTACACAGAGCACACAGCC	56	746	[30]
3	*Cytauxzoon felis*	F: CCAGCTCCAATAGCGTATATT R: AGGATGAACTCGATGAATGCA	61	431	[31]
4	*Hepatozoon felis*	F: CTTACCGTGGCAGTGACGGT R: TGTTATTTCTTGTCACTACCTCTCTTATGC	58	146	[32]
5	*Anaplasma platys*	F: GATTTTTGTCGTAGCTTGCTATG R: TAGCACTCATCGTTTACAGC	55	678	[33]
6	*A. phagocytophilum*	F: ATGAATTACAGAGAATTGCTTGTAGG R: TTAATTGAAAGCAAATCTTGCTCCTATG	54	849	[34]
7	*Borrelia burgdorferi*	F: AATAGGTCTAATATTAGCCTTAATAGC R: TCAAGTCTGGTTCCGTCTGCTC	60	417	[35]
8	*Bartonella henselae*	F: TTCCGYCTTATGGGTTTTGG R: CATTTCTGTTGGAAATCCTAG	52	246	[12]
9	*Rickettsia felis*	F: CCGATTCAGCAGGTTCTTCAA R: ATGTTCGGGCTTCCGGTATG	57	120	[36]
10	Hemotropic *Mycoplasma spp*	F: GCCCATATTCCTACGGGAAGCAGCAGT R: CTCCACCACTTGTTCAGGTCCCCGTC	68	620	[21]
11	*Plasmodium spp*	F: CCTGTTATCCCCGGCGAACCTTC R: CTCGCCATTTGATAGCGGTTAACC	63	937	[37]
12	*Leishmania* *donovani*	F: GGCATAAATCCATGTAAGA R: TGGCTTTTATATTATCATTTT	54	540	[38]
13	*Ehrlichia chaffeensis*	F: AGATACTTCAAGCTCTATTC R: AGGTAGTGGTATTAACGG	49	277	[39]
14	*Neoehrlichia mikurensis*	F: AACAGGTGAAACACTAGATAAGTCCAT R: TTCTACTTTGAACATTTGAAGAATTACTAT	58	950	[40]

**Table 2:** The prevalence of identified pathogens in cats.

Pathogens	Frequency				
	−	+	+ (Row %)	*χ* ^2^	*p* Value
*Babesia microti*	163	4	2.4	152.26	<0.001
Adjusted residual (z-score)	5.5	−5.5			
*p* value *	<0.001	<0.001			
*Babesia canis canis*	127	40	24.0		
Adjusted residual (z-score)	−2.1	2.1			
*p* value *	0.036	0.036			
*Cytauxzoon felis*	156	11	6.6		
Adjusted residual (z-score)	4.1	−4.1			
*p* value *	<0.001	<0.001			
*Hepatozoon felis*	149	18	10.8		
Adjusted residual (z-score)	2.6	−2.6			
*p* value *	0.009	0.009			
*Anaplasma phagocytophilum*	155	12	7.2		
Adjusted residual (z-score)	3.8	−3.8			
*p* value *	<0.001	<0.001			
*Anaplasma platys*	116	51	30.5		
Adjusted residual (z-score)	−4.4	4.4			
*p* value *	<0.001	<0.001			
*Rickettsia felis*	123	44	26.3		
Adjusted residual (z-score)	−2.9	2.9			
*p* value *	0.004	0.004			
*Bartonella henselae*	100	67	40.1		
Adjusted residual (z-score)	−7.8	7.8			
*p* value *	<0.001	<0.001			
*Borrelia burgdorferi*	132	35	21.0		
Adjusted residual (z-score)	−1.0	1.0			
*p* value *	0.3	0.3			
Hemotropic *Mycoplasma*	148	19	11.4		
Adjusted residual (z-score)	2.4	−2.4			
*p* value *	0.02	0.02			

* If *p*-value < 0.0025 (Bonferroni-corrected *p* value) then statistically significant. New critical z-score = −3.02.

**Table 3:** The prevalence of identified pathogens in cats by gender.

	Frequency			*χ* ^2^	*p* Value	Fisher′s Exact Test *p* Value
Group	–	+	+ (Row %)			
	*Babesia microti*					0.362
Male	84	1	1.2			
Female	79	3	3.7			
	*Babesia canis canis*			3.78	0.052	
Male	70	15	17.6			
Female	57	25	30.5			
	*Cytauxzoon felis*			0.14	0.709	
Male	80	5	5.9			
Female	76	6	7.3			
	*Hepatozoon felis*			1.16	0.281	
Male	78	7	8.2			
Female	71	11	13.4			
	*Anaplasma phagocytophilum*			3.47	0.062	
Male	82	3	3.5			
Female	73	9	11.0			
	*Anaplasma platys*			1.05	0.307	
Male	56	29	34.1			
Female	60	22	26.8			
	*Rickettsia felis*			0.318	0.573	
Male	61	24	28.2			
Female	62	20	24.4			
	*Bartonella henselae*			1.68	0.195	
Male	55	30	35.3			
Female	45	37	45.1			
	*Borrelia burgdorferi*			3.35	0.067	
Male	72	13	15.3			
Female	60	22	26.8			
	Hemotropic *Mycoplasma*		10.6	0.11	0.744	
Male	76	9	12.2			
Female	72	10				

**Table 4:** The prevalence of identified pathogens in cats by age group.

	Frequency			*χ* ^2^	*p* Value	Fisher′s Exact Test *p* Value
Group	−	+	+ (Row %)			
	*Babesia microti*					1.000
<1 year	77	2	2.5			
>1 year	86	2	2.3			
	*Babesia canis canis*			3.20	0.074	
<1 year	65	14	17.7			
>1 year	62	26	29.5			
	*Cytauxzoon felis*			1.90	0.169	
<1 year	76	3	3.8			
>1 year	80	8	9.1			
	*Hepatozoon felis*			0.55	0.458	
<1 year	69	10	12.7			
>1 year	80	8	9.1			
	*Anaplasma phagocytophilum*			**7.88**	**0.005**	
<1 year	78	1	1.3			
>1 year	77	11	12.5			
	*Anaplasma platys*			0.94	0.333	
<1 year	52	27	34.2			
>1 year	64	24	27.3			
	*Rickettsia felis*			0.08	0.774	
<1 year	59	20	25.3			
>1 year	64	24	27.3			
	*Bartonella henselae*			0.28	0.592	
<1 year	49	30	38.0			
>1 year	51	37	42.0			
	*Borrelia burgdorferi*			0.95	0.330	
<1 year	65	14	17.7			
>1 year	67	21	23.9			
	Hemotropic *Mycoplasma*			0.23	0.630	
<1 year	71	8	10.1			
>1 year	77	11	12.5			

**Table 5:** The prevalence of identified pathogens in cats by gender and age group.

	Frequency			*χ* ^2^	*p* Value
Group	−	+	+ (Row %)		
Male	714	136	16.0	4.80	0.028
Female	655	165	20.1		
<1 year	661	129	16.3	2.92	0.088
>1 year	708	172	19.5		

**Table 6:** Spearman’s rank correlation coefficients (rho) between the identified pathogens in cats.

	Bm	Bcc	Cf	Hf	Ap	Apl	Rf	Bh	Bb	HM
Bm	1.000	**0.279**	0.116	−0.054	0.108	0.151	−0.094	0.111	0.112	0.067
	*p* value	**<0.001**	0.135	0.485	0.165	0.051	0.228	0.151	0.150	0.388
Bcc		1.000	0.134	0.031	0.061	0.115	0.047	0.170^*^	**0.228**	−0.024
	*p* value		0.085	0.689	0.432	0.138	0.550	0.028	**0.003**	0.755
Cf			1.000	−0.092	**0.207**	0.034	0.060	0.029	0.041	0.057
	*p* value			0.236	**0.007**	0.667	0.438	0.711	0.597	0.465
Hf				1.000	−0.022	0.021	0.055	0.109	0.011	−0.003
	*p* value				0.778	0.787	0.479	0.159	0.890	0.970
Ap					1.000	0.017	−0.061	0.103	0.028	−0.027
	*p* value					0.829	0.432	0.184	0.723	0.732
Apl						1.000	0.046	**0.253**	0.010	0.049
	*p* value						0.554	**0.001**	0.898	0.529
Rf							1.000	0.037	0.160^*^	**0.171**
	*p* value							0.632	0.039	**0.027**
Bh								1.000	**0.239**	–0.024
	*p* value								**0.002**	0.759
Bb									1.000	–0.046
	*p* value									0.559
HM										1.000

Bm = *Babesia microti*. Bcc = *Babesia canis canis*. Cf = *Cytauxzoon felis*. Hf = *Hepatozoon felis*. Ap = *Anaplasma phagocytophilum*. Apl = *Anaplasma platys*. Rf = *Rickettsia felis*. Bh = *Bartonella henselae*. Bb = *Borrelia burgdorferi*. HM = *Hemotropic Mycoplasma*.

## Data Availability

All relevant data are provided in the manuscript. Raw data can be made available on reasonable demand.
